# Effect of Adding Losartan to Bevacizumab for Treating Diabetic Macular Edema

**DOI:** 10.1155/2020/4528491

**Published:** 2020-10-01

**Authors:** Fariba Ghassemi, Abdulrahim Amini, Masoud Yasemi, Amin Nabavi, Mohammadkarim Johari

**Affiliations:** ^1^Department of Ophthalmology, Ophthalmology Research Center, Farabi Eye Hospital, Tehran University of Medical Sciences, Tehran, Iran; ^2^Hormozgan University of Medical Sciences, Bandar Abbas, Iran; ^3^Poostchi Ophthalmology Research Center, Department of Ophthalmology, School of Medicine, Shiraz University of Medical Sciences, Shiraz, Iran; ^4^Eye Research Center, Farabi Eye Hospital, Tehran University of Medical Sciences, Tehran, Iran

## Abstract

**Introduction:**

Diabetic retinopathy is the most common cause of visual loss and blindness in the age group of 20 to 64 years. This study aimed to evaluate the efficacy of oral Losartan adjuvant therapy in combination with intravitreal injection of Bevacizumab in the treatment of diabetic macular edema.

**Methods:**

In this randomized clinical trial, 61 eyes of 47 patients with normal blood pressure and diabetic macular edema and nonproliferative diabetic retinopathy were studied. Patients were randomly divided into Losartan (*n* = 33) and control (*n* = 28) groups. All patients received 3–6 intravitreal injections of Bevacizumab over 6 months. General examination including blood pressure and glycosylated hemoglobin measurements were performed in all patients. Complete ophthalmologic examination and macular OCT were performed at the first, third, and sixth months of treatment in all patients.

**Results:**

The mean age of the patients studied was 57.1 ± 7.4 years and 37.7% of the patients were male. There was no significant difference between the two groups in terms of initial visual acuity, central macular thickness, and frequency of injections. There was no significant difference in visual acuity and central macular thickness between the two groups at the first, third, and sixth months of treatment. Age, frequency of injection, and initial macular thickness less than 450 microns were effective in patients' final visual acuity.

**Conclusion:**

Short-term adjuvant treatment with Losartan in patients with diabetic macular edema and nonproliferative diabetic retinopathy has no greater effect than the standard treatment.

## 1. Introduction

According to the reports provided by the World Health Organization (WHO) in January 2011, more than 220 million people worldwide had diabetes, and it will reach 366 million by 2030 [1]. Diabetic retinopathy is the most common complication of diabetes and the leading cause of blindness in people of working age. The disease can be either nonproliferative or proliferative. Macular edema can occur at any stage of the disease. The most common manifestation of ocular involvement in type 2 diabetes is macular edema [2, 3]. Although the pathogenesis of diabetic retinopathy is not fully understood, its major risk factors include hyperglycemia and hypertension. One of the factors that have recently been implicated more prominently in the pathogenesis of diabetic retinopathy is the renin-angiotensin system (RAS). The RAS is a major contributor to the microvascular complications of diabetes that cause numerous tissue responses including vasoconstriction, inflammation, oxidative damage, neovascularization, and fibrosis [4]. According to the literature, angiotensin II increases the exertion of exudates from the retinal vessels [5]. Angiotensin II can also stimulate the formation of new retinal vessels by increasing the activity of the vascular endothelial growth factor (VEGF) and other growth factors. The studies in animal models have shown protective effects of RAS inhibitors in the retina [6]. Numerous studies on RAS components in the retina have shown increased levels of prorenin, renin, and angiotensin II in the vitreous of the patients with proliferative diabetic retinopathy (PDR) and diabetic macular edema (DME), suggesting the essential role of RAS in the pathogenesis of diabetic retinopathy [7, 8]. Also, the RAS drug block at the level of angiotensin-converting enzyme (ACE) or angiotensin receptors decreases intraretinal angiogenesis and decreases vascular leakage, which is the cause of macular edema [9, 10].

There have been many treatments considered for diabetic macular edema (DME) so far. These treatments include macular laser photocoagulation, intravitreal injection of antivascular endothelial growth factor (Anti-VEGF) compounds, intravitreal injection of corticosteroids, and other therapies [11–13]. Given the aggressive nature of these treatments and their potential side effects, as well as the failure of a group of patients to respond to these treatments, followed by the need for repeating the treatment, there is a need for alternative or complementary therapies. Given the proven role of the renin-angiotensin system in the development of diabetic macular edema, the availability of low-cost oral systemic drugs, and relatively few complications to block this system, the use of angiotensin receptor blockers (ARBs) such as Losartan simultaneously with intravitreal injection of Anti-VEGF compounds, including Bevacizumab, which is one of the most effective therapies currently available, may improve the clinical response to these drugs and reduce the frequency of injections. The purpose of this study was to evaluate the efficacy of oral Losartan adjuvant therapy in combination with intravitreal injection of Bevacizumab in the treatment of diabetic macular edema.

## 2. Materials and Methods

### 2.1. Study Setting and Ethical Considerations

For doing this double-blind placebo-controlled randomized clinical trial, a total of 79 eyes of 52 patients were studied, and finally, 61 eyes of 42 patients completed the study. The information related to the patients in 2017-2018 was collected in the ophthalmology clinic of Tehran University of Medical Sciences (TUMS). This research project was approved by the ethics committee of TUMS and the patients granted informed consent before inclusion based on the Helsinki Declaration. The ocular examinations included visual acuity measurement (based on LogMAR), slit lamp examination, intraocular pressure measurement, and retinal examination after the dilation of the pupil with Tropicamide (1% Tropicamide, Sina Darou). Patients underwent paraclinical eye examinations including spectral domain-optical coherence tomography (SD-OCT), macular examination, and fluorescein angiography (FA). Blood pressure was measured in all patients. All patients had fasting blood sugar (FBS), HbA1C, complete blood count (CBC), urea, creatinine, triglyceride, low-density lipoprotein (LDL), high-density lipoprotein (HDL), cholesterol, and serum potassium and albumin measurements.

### 2.2. Patient Enrollment and Follow-up

The patients were divided into two groups. The first group was treated with intravitreal injection of Bevacizumab (1.25 mg) and oral Losartan (50 mg) every day. The second group was treated with intravitreal injection of Bevacizumab (1.25 mg) and placebo (vitamin C 100 mg). All factors were randomly divided into two intervention and nonintervention groups using the block randomization method. Patients with type 1 and 2 diabetes were allowed for inclusion if they were normoalbuminuric and normotensive (blood pressure 130/85 mm Hg or below), also only patients with moderate to severe nonproliferative diabetic retinopathy were enrolled.

Patients treated with Losartan were told about the complications and were advised to visit the relevant physician in case of any complication. One week after treatment, blood pressure was remeasured and the drug was discontinued if severe hypotension occurred. The patients were asked about drug-related complications on all visits. All patients were followed up at 1, 3, and 6 months intervals. All examinations performed during all the visits included visual acuity measurement, fundoscopy, macular thickness measurement based on macular OCT, fluorescein angiography, as well as the measurement of fasting blood sugar (FBS), HbA1C, and blood pressure levels. Macular thickness was assessed by spectral domain-optical coherence tomography (SD-OCT). For this purpose, Spectralis HRA-OCT version 5.3.3.0 (Heidelberg Co, Germany) was used. Before imaging, pupils were dilated with 1% Tropicamide. The patient was asked to look at the marker light for appropriate fixation. The retina image was shown on the screen, and after proper focus, it was recorded on the macula area by the camera. The central macular thickness was reported in microns.

### 2.3. Statistical Analysis

To analyze the data, in the descriptive statistics part, the statistical indices including mean for quantitative variables and frequency/percentage for qualitative variable were used for the study population. The *t*-test was used to compare the quantitative variables such as macular thickness. To compare the qualitative variables such as the frequency of injections, the chi-square test was used. A 95% confidence level was considered in statistical tests. Finally, data analysis was performed using SPSS version 21.

## 3. Results

The study began with the examination of 79 eyes of 52 patients, but eventually 61 eyes of 42 patients completed the study. One patient in the case group died during the follow-up. Another patient in this group was excluded because of hypotension. 3 eyes of 2 patients (2 eyes in the control group and 1 eye in the case group) advanced to proliferative diabetic retinopathy (PDR) and were excluded from the study. Eleven patients did not complete the follow-up for 6 months and were not included in the analysis. Consequently, a total of 61 eyes were evaluated in both case (33 eyes) and control (28 eyes) groups. The mean age of participants was 57.14 ± 7.42 years. The youngest studied was 25 years and the oldest was 72 years old. 37.7% of the study participants (23 individuals) were female and 62.3% (38 individuals) were male. There was no significant difference in age and sex between the two groups. The data on the disease stage and associated laboratory results of the patients are summarized in Table 1. There was no significant difference between the two groups in terms of diabetic retinopathy, duration of diabetes, and metabolic control data including FBS and HbA1c.

**Figure 1:**
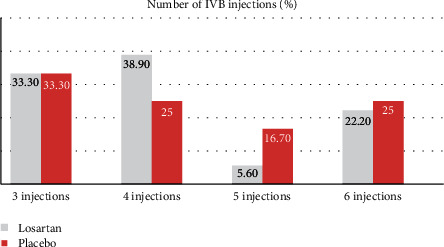
Comparison of the number of injections between the two study groups during the follow-up period (*p*=0.76).

There was no significant difference in best-corrected visual acuity (BCVA) and macular thickness between the two groups before the treatment. Patients in both groups received 3–6 Bevacizumab injections over 6 months.  Figure 1 shows the details of frequency of injection in the two groups under study. The chi-square test showed no significant difference between the two groups in the frequency of injection (*P*=0.76).

Before the intervention, BCVA was 0.47 ± 0.32 in the case group and logMAR 0.45 ± 0.26 in the control group (*p*=0.86). There was no significant difference in BCVA before the intervention between the two control and case groups. No significant difference in BCVA was observed between the eyes of the case and control groups on any of the follow-up visits at the first, third, and sixth months (*p* > 0.05 in all cases) (Table 2).

The mean macular thickness in OCT before the intervention was 452.6 ± 116.8 and 443.5 ± 101.4 microns for patients in the case and control groups, respectively, and no significant difference was found between the two groups (*p*=0.52). Like BCVA, there was no significant difference between the eyes of two groups in terms of macular thickness on any of the follow-up visits (Table 3).

**Figure 2:**
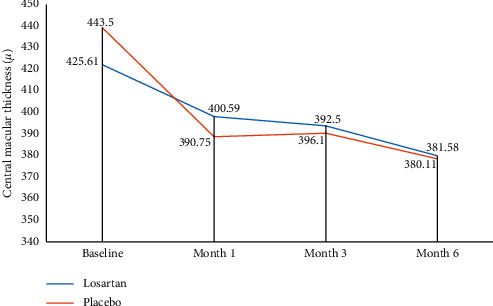
Central macular thickness changes in two studied groups during six months.

Changes in central macular thickness in the two study groups during follow-up are shown in Figure 2.

The Pearson test was used to investigate the affecting factors on BCVA recovery after 6 months. According to Table 4, the frequency of injection and age were significantly associated with the rate of final BCVA improvement. Initial macular thickness was close to a significant level (*p*=0.064). Dividing the patients into two groups with initial macular thickness less than and more than 450 microns showed that the effect of treatment was higher in patients with a less initial macular thickness (*p*=0.03). At the last follow-up time, a total of 4 eyes of 3 patients in the control group and 5 eyes of 3 patients in the case group progressed from moderate to severe NPDR. The rate of diabetic retinopathy progression was 14.2% in the control group versus 15.1% in the case group which was not significant between the two groups (*p*=0.12).

Also, in this study, no significant complications of intravitreal injection including endophthalmitis and traumatic cataract were observed in either group. Hypotension episodes were only observed in one patient among those who treated with Losartan and the patient was excluded.

## 4. Discussion

The renin-angiotensin system (RAS) is a hormone system that regulates blood pressure and water balance. Increased RAS activity causes cardiac complications and the progression of diabetic nephropathy. In addition to affecting hypertension, such complications have been attributed to local effects such as thrombosis, fibrosis, inflammation, and oxidation [4–6]. According to some studies, in addition to the indirect effects of the system, the possibility of localized RAS activity in the eye has also been raised, so the oral use of drugs inhibiting this system for reducing the ocular complications of diabetes has been taken into account [14]. However, clinical trials on the clinical impact of RAS inhibitors on the progression of diabetic retinopathy have conflicting results. In a 2-year, double-blind clinical trial, Chaturvedi et al. investigated the effect of angiotensin-converting enzyme (ACE) inhibitor, Lisinopril, on the progression of diabetic retinopathy in type 1 diabetic patients with normal blood pressure. In this study, the drug decreased the progression of diabetic retinopathy in patients with type 1 diabetes and slowed the progression of nonproliferative diabetic retinopathy toward proliferative diabetic retinopathy [6]. In another study, Sjølie et al. investigated the effect of Candesartan on the progression and regression of diabetic retinopathy in patients with type 2 diabetes. In this large study, 1905 patients were divided into two groups of drug or placebo recipients. Based on the results of this study, the rate of progression of diabetic retinopathy in the case group was not significantly decreased compared to the control group. The rate of regression reported for the patients in the case group was 34% compared to the control group, which was significant (*p*=0.009). The treatment complication rate was similar in both groups. However, in this study, the rate of progression of diabetic retinopathy toward the proliferative stage and the incidence of macular edema was not significantly different between the two groups [15].

In contrast to these studies, other studies do not confirm the effective role of these adjuvant therapies. In the study of Pradhan et al., 35 patients with type 2 diabetes and normal blood pressure who had not been treated with angiotensin-converting enzyme inhibitor (ACE inhibitors) drugs so far were evaluated. In this study, low doses of Enalapril did not play a role in preventing the progression of diabetic retinopathy [4]. Pradhan et al. did not observe any changes in the rate of diabetic maculopathy in patients treated with Losartan during the 4-month follow-up of patients [4]. According to the results of our study, the use of Losartan adjunctive therapy for diabetic macular edema (DME) was not more effective than the standard treatment (intravitreal injection of antivascular endothelial growth factor (Anti-VEGF)). However, it should be noted that different studies on the effect of renin-angiotensin system (RAS) blockers on diabetic retinopathy have major differences with each other and also with the current study. One of the most important differences in these studies is the treatment goal. In the large study of Sjølie et al., Candesartan reduced the progression of diabetic retinopathy in cases with mild to moderate nonproliferative retinopathy [15], but the beneficial effect of this drug in this study was only limited to this case and did not affect the incidence rate of macular edema as in our study. Also, some of these studies which are similar to our study excluded hypertensive patients and others evaluated these patients. In the study of Sjølie et al., both groups of nonhypertensive and hypertensive patients treated were evaluated [15]. In hypertensive patients treated, blood pressure lower than 100/160 was considered a criterion. It seems that the inclusion of hypertensive patients may hamper the assessment of local or independent effects of hypertensive control of renin-angiotensin system blockers. In fact, hypertension is a well-known risk factor for the progression of diabetic retinopathy and the development of its associated complications, and the control of blood pressure with any drug can be effective in preventing these complications.

In our study, only one case of hypotension episodes was observed in one of the patients treated with Losartan. In the study of Sjølie et al., complications observed with Candesartan that discontinued the treatment occurred in 4% of the patients in the case group and 4% in the control group in total [15]. Due to the use of Captopril, 2 patients with lacunar stroke and 10 patients with chronic cough were reported in the study by Wang et al. [16].

## 5. Conclusion

The use of Losartan adjuvant therapy in our study had no additional effect on the treatment of diabetic macular edema. However, further studies with a larger sample size and longer follow-up should be undertaken to investigate this issue.

## 6. Limitations

The most important limitation of this study was the short duration of follow-up and sample size. Besides, we did not use the same placebo in this study and applied vitamin C ( 00 mg) as placebo; although it was a really minimal dose to have any effect, some studies showed vitamin C in dosing of 2000 mg may have protective effect in progression of proliferative diabetic retinopathy.

## Figures and Tables

**Table 1:** Comparison of clinical and laboratory data of the case and control groups.

Variable	Case group (*n*=33)	Control group	*p* value
Stage of diabetic retinopathy			0.62^*∗*^
Moderate NPDR	11 (33.33%)	6 (21.5%)	
Severe NPDR	22 (66.7%)	22 (78.5%)	

Period of time	9.12 ± 4.26	11.15 ± 4.36	0.11^*∗∗*^
HbA1c (mg/dl)	208.1 ± 89.6	172.5 ± 65.4	0.08^*∗∗*^
FBS (mg/dl)	8.51 ± 1.66	8.11 ± 1.45	0.48^*∗∗*^

^*∗*^ Chi-square test. ^*∗∗*^*T*-test.

**Table 2:** Comparison of BCVA results of two groups after first, third, and sixth months follow-up.

Best-corrected visual acuity (BCVA)#	Case group (*n* = 33)	Control group	*p* value^*∗*^
Before intervention	0.47 ± 0.32	0.45 ± 0.26	0.86
One month after intervention	0.40 ± 0.31	0.37 ± 0.22	0.71
Three months after intervention	0.33 ± 0.26	0.36 ± 0.24	0.66
Six months after intervention	0.27 ± 0.26	0.25 ± 0.23	0.68

^*∗*^ *T*-test. ^#^Based on Log MAR

**Table 3:** Comparison of macular thickness between two groups before and after intervention.

Macular thickness^#^	Control group	Case group	*p* value^*∗*^
Before intervention	45.2 ± 116.8	443.5 ± 101.4	0.52
1 month after intervention	400.5 ± 121.7	390.7 ± 91.4	0.75
3 months after intervention	396.1 ± 120.8	392.5 ± 131.4	0.92
6 months after intervention	381.5 ± 124.5	380.1 ± 127.5	0.96

^*∗*^ *T*-test. ^#^Based on micrometer

**Table 4:** Result of multivariate linear regression analysis for evaluation of the variable effects on improvement of best-corrected visual acuity (BCVA)

*p* value	*r*	Variable
0.006	0.348	Age
0.65	−0.06	Duration of diabetes
0.043	0.145	Frequency of injection
0.064	−0.241	Macular thickness before intervention
0.9	−0.01	HbA1c

## Data Availability

The data used to support the findings of this study are available from the corresponding author upon request.
